# Glutamine Metabolism in Brain Tumors

**DOI:** 10.3390/cancers11111628

**Published:** 2019-10-24

**Authors:** Siva Kumar Natarajan, Sriram Venneti

**Affiliations:** 1 Laboratory of Brain Tumor Metabolism and Epigenetics, Department of Pathology, University of Michigan Medical School, Ann Arbor, MI 48109, USA; sivakn@umich.edu; 2 Department of Pathology, University of Michigan 3520E MSRB 1, 1150 West Medical Center Drive, Ann Arbor, MI 41804, USA

**Keywords:** brain tumor, metabolism, glutamine, glutamate, redox homeostasis, imaging, therapy

## Abstract

Altered metabolism is a hallmark of cancer cells. Tumor cells rewire their metabolism to support their uncontrolled proliferation by taking up nutrients from the microenvironment. The amino acid glutamine is a key nutrient that fuels biosynthetic processes including ATP generation, redox homeostasis, nucleotide, protein, and lipid synthesis. Glutamine as a precursor for the neurotransmitter glutamate, and plays a critical role in the normal functioning of the brain. Brain tumors that grow in this glutamine/glutamate rich microenvironment can make synaptic connections with glutamatergic neurons and reprogram glutamine metabolism to enable their growth. In this review, we examine the functions of glutamate/glutamine in the brain and how brain tumor cells reprogram glutamine metabolism. Altered glutamine metabolism can be leveraged to develop non-invasive imaging strategies and we review these imaging modalities. Finally, we examine if targeting glutamine metabolism could serve as a therapeutic strategy in brain tumors.

## 1. Introduction

Brain tumors in adults and children are challenging tumors to treat. Aggressive gliomas including glioblastomas (GBM) and high-grade gliomas have very poor outcomes, emphasizing the need to better understand their pathogenesis. The field of neuro-oncology has seen significant strides from the perspective of identifying genetic drivers of these cancers. Next generation sequencing has revealed distinct mutations in adult and childhood gliomas. Many of these genetic mutations, including alterations in receptor kinase signaling (including epidermal growth factor receptor (EGFR) amplifications and mutations), mammalian target of rapamycin (mTOR) [through both receptor tyrosine kinase mutations and PTEN (phosphatase and tensin homolog) deletion], MYC activation and isocitrate dehydrogenase (IDH) mutations directly impact metabolic pathways, including glutamine metabolism. Cancer cells can use nutrients such as glucose and the amino acid glutamine to support continuous proliferation and macromolecule biosynthesis. These metabolic needs can be regulated by cell-intrinsic mechanisms as well as nutrient availability in the tumor microenvironment [1]. In this context, the microenvironment of the brain is unique because of its glutamine and glutamate rich environments. The brain tumor microenvironment consists of neurons, glial cells including microglia, astrocytes, oligodendrocytes, endothelial cells, infiltrating T-cells, and tumor associated macrophages. While it is known that tumor cells in general can be metabolically coupled to cells in the microenvironment, it is not known if and how brain tumor cells are metabolically linked to each of these cells.

The brain accounts for approximately 25% of glucose consumed by the human body [2]. Important cerebral functions like maintaining ion gradients and synaptic excitability necessitate such high energy demands. While glucose serves as an obligatory energy substrate of the brain, blood-derived substrates like ketone bodies and glutamine can also be utilized by the brain in varying capacities. Glutamine is the most abundantly available amino acid in the blood, with extracellular fluid concentrations reaching up to 472 ± 38 μM in the brain [3]. Along with glucose, glutamine serves as the primary precursor for synthesis of glutamate, a critical excitatory neurotransmitter. From the perspective of glutamine metabolism and the brain tumor microenvironment, the normal functions of neurons and glial cells is critical to understand. In this review, we focus on how brain tumor cells utilize glutamine in the context of this unique microenvironment, and some of the limitations we encounter in studying glutamine metabolism. From a translational point of view, non-invasive glutamine imaging can be used as a means to monitor brain tumors. Furthermore, we examine the potential of targeting glutamine metabolism in brain tumors as novel therapeutic avenues to treat these tumors.

## 2. Glutamate/Glutamine Metabolism in the Central Nervous System

### 2.1. Synaptic Glutamate/Glutamine Cycle is Critical for Brain Function

The amino acid glutamate serves as an important excitatory neurotransmitter, and neurons that release synaptic glutamate are termed glutamatergic neurons. Main glutamatergic pathways connecting various brain regions include glutamatergic projections from the cortex to the brainstem, from the prefrontal cortex to the striatal part of the basal ganglia (corticostriatal pathway), and projections to and from the thalamus to the cortex. Additionally, pyramidal neurons within the cortex can communicate with each other through glutamatergic connections. Similarly, glutamatergic neurons in the cerebellum can communicate with other cerebellar neurons. Because of the nature of these specific projections, glutamate and subsequently glutamine concentrations can vary depending on the brain region, with cortical and cerebellar regions having the highest concentrations. These inter-regional differences in the milieu have implications for the amount of glutamine available for tumor cells.

**Figure 1:**
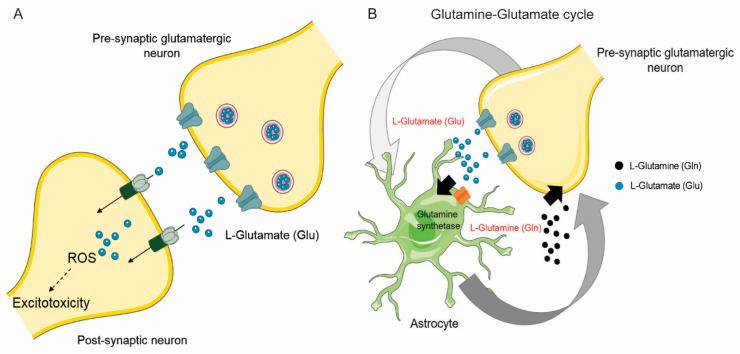
Glutamine/Glutamate neuroglial cycle and glutamate-induced excitotoxicity. (**A**) Pre-synaptic glutamatergic neurons utilize glutamine to synthesize the excitatory neurotransmitter, glutamate, which is then released during synaptic transmission. Glutamate then interacts with post-synaptic glutamatergic receptors. Excess glutamate however, leads to oxidative stress and neuronal injury, termed as excitotoxicity. (**B**) Astrocytes help mitigate glutamate-induced excitotoxicity by removing glutamate from the synapse. Glutamate is then converted to glutamine by the enzyme glutamine synthetase, an enzyme that is present in astrocytes but not in neurons. Glutamine is recycled to neurons to enable glutamate synthesis. Abbreviations: ROS, reactive oxygen species.

Glutamatergic neurons release glutamate into the synaptic cleft through vesicular exocytosis. This glutamate is essential for synaptic function to regulate memory formation and normal cognitive function. Subsequently, glutamate is taken up by astrocytes by astrocytic glutamate transporters and is synthesized to glutamine [2,3]. Glutamine is then transported to neurons, where it is converted back to glutamate for synaptic transmission [4,5,6]. This is termed the glutamine-glutamate cycle (Figure 1). While it is known that glutamate is essential for several critical functions in the central nervous system (CNS), excess accumulation of glutamate can be detrimental to normal brain function, resulting in neuronal excitotoxicity and cell death [7]. Excitotoxicity plays an important role in the pathogenesis of seizures, stroke, and neurodegeneration [8,9]. Astrocytes play a key role in maintaining glutamate homeostasis by regulating its synthesis from glucose as well as its recycling from the synapse. They are one of the principle components of the brain cytoarchitecture, and mitigate glutamate toxicity by the glutamate/glutamine neuroglial cycle [5], along with other critical functions. Indeed, more than 70% of synaptic glutamate is derived from the glutamate/glutamine cycle [10,11]. Astrocytes accomplish this by increasing glutamate uptake from the synapse and converting it back into glutamine through the astrocyte-specific enzyme glutamine synthetase (GS). Glutamine is then recycled back to neurons. Glutamate uptake in astrocytes is mediated through different classes of glutamate transporters that are functionally distinct.

### 2.2. Neurons Express Glutamatergic Receptors That Mediate Synaptic Signaling

Once glutamate is released into the synaptic cleft, it interacts with specific glutamatergic receptors expressed in postsynaptic neurons. Post synaptic glutamate receptors are classified into three different families, with each serving a distinct purpose [12]. These include N-methyl-D-aspartate (NMDA), α-amino-3-hydroxy-5methyl-4-isoxazole propionic acid (AMPA), and metabotropic glutamatergic receptors. NMDA receptors are tetrameric assemblies of ionotropic receptor subunits. They are directly involved in synaptic transmission and plasticity, and are essential for learning and formation of memory [13]. NMDA receptors are ligand-gated ion channels which become permeable to Na^+^ and Ca^2+^ ions upon glutamate binding. They consist of five receptor subunits (GluN1 and GluN2A-D) and a pair of GluN3 subunits (GluN3A and GluN3B), wherein each subunit has a specific function depending on their composition [14,15]. All receptors consist of two copies of GluN1 along with two of GluN2 and/or GluN3 subunits. The composition of subunits impacts the receptor’s biophysical, pharmacological, and signaling attributes. For example, the NR1 subunit is essential for formation of a functional receptor and mediates binding of glycine [16]. Whereas, NR2 receptors increase the influx of Ca^2+^ ions upon glutamate binding, which in turn activates downstream signaling mechanisms involving protein kinase A (PKA) and CAMKII [17]. It is noteworthy that this interaction of GluN2B and CAMKII is critical for regulation of neuronal survival, synapse formation, and plasticity [18,19].

The AMPA receptor family consists of four subunits (GluR1-4) with various combinations necessary to form a functional ionotropic channel [20]. They activate and desensitize more rapidly than NMDA receptors, thus facilitating faster synaptic transmission [21]. The cation permeability of the channel is determined by AMPA receptor subunit, mainly, GluR2. AMPARs that lack GluR2 are permeable to both Na^+^ and Ca^2+^, whereas a channel that lacks GluR2 will be impermeable to Ca^2+^ [22].

Metabotropic glutamatergic (mGluRs) receptors are G-protein-coupled receptors that mediate slower glutamatergic responses. These receptors are expressed in both pre- and post-synaptic terminals, and are primarily involved in regulation of synaptic transmission [23]. They modulate NMDA and AMPA receptors by regulating glutamate release into the synapse, thereby affecting synaptic plasticity [24,25]. These receptors have eight subtypes (mGluR 1–8) and are often coupled to the phospholipase C/inositol triphosphate/diacylglycerol pathway. The function of these receptors can be negatively regulated by the adenylate cyclase pathway [23].

### 2.3. Astrocytes Take Up Glutamate through Excitatory Amino Acid Transporters (EAATs)

Astrocyte processes extending into the synaptic cleft abundantly express glutamate transporters, which serve to rapidly remove glutamate from the synapse. Sodium dependent excitatory amino acid transporters (EAATs) are the primary class of glutamate transporters involved in glutamate homeostasis. There are five main subtypes of high affinity Na^+^ -dependent EAATs: EAAT1/GLAST, EAAT2/GLT1, EAAT3/EAAC1, EAAT4, and EAAT5. These transporters are Na^+^ and K^+^- dependent and co-transport L- and D- aspartate along with L-glutamate. EAAT3 and EAAT4 are particularly expressed in certain Purkinje cells, while EAAT5 is expressed in retinal cells [26,27]. EAAT1 and EAAT2 are primarily astrocytic, with EAAT2 abundantly expressed glutamate transporter accounting for 95% of forebrain glutamate uptake activity [28,29].

### 2.4. System Xc- and Sodium-Coupled Neutral Amino Acid Transporters (SNATs) can Regulate the Glutamine Glutamate Cycle

Extracellular glutamate levels are also influenced by the Na^+^ -independent, cystine/glutamate exchanger, system x_c_ (SXC). SXC is a Cl^−^- dependent glutamate transporter that is expressed predominantly in astrocytes, oligodendrocytes, and some cortical neurons [30,31]. It belongs to the heteromeric amino acid transporter (HAT) family, with a heavy subunit CD98 and lighter subunit coupled through disulfide bridges. SXC plays an important role in regulating redox balance within the cell as it exchanges glutamate for cystine, which in turn is converted to cysteine. This is the rate-limiting step for the synthesis of intracellular glutathione (GSH), the primary intracellular anti-oxidant in the CNS. High levels of glutamate can result in production of reactive oxygen species (ROS) that causes protein carbonylation and lipid peroxidation, resulting in cell death. Oxidative stress or electrophiles induce expression of SXC to synthesize GSH, which in turn scavenges ROS.

Astrocytes recycle glutamate and convert it into glutamine, which is then released into the extracellular space through the system N or sodium-coupled amino acid transporters (SNATs). SNAT3/SLC38A3 and SNAT5/SLC38A5 play key roles in mediating bidirectional, electroneutral transport of glutamine in astrocytes [32,33]. They are also involved in co-transport of other amino acids like alanine, histidine, asparagine, and glycine [34]. The importance of maintaining a balance between extracellular levels of glutamate and glutamine necessitate coordination in glutamate uptake and glutamine release by astrocytes. Indeed, there is evidence for physical coupling between EAAT1 and SNAT3/SLC38A3 in astrocytes [35]. This suggests that astrocytes are dependent on these transporters to sense extracellular release of glutamate and respond by recycling it to release glutamine.

### 2.5. Brain Tumor Cells can Promote Their Growth Via Glutamine/Glutamate Transporters

**Figure 2:**
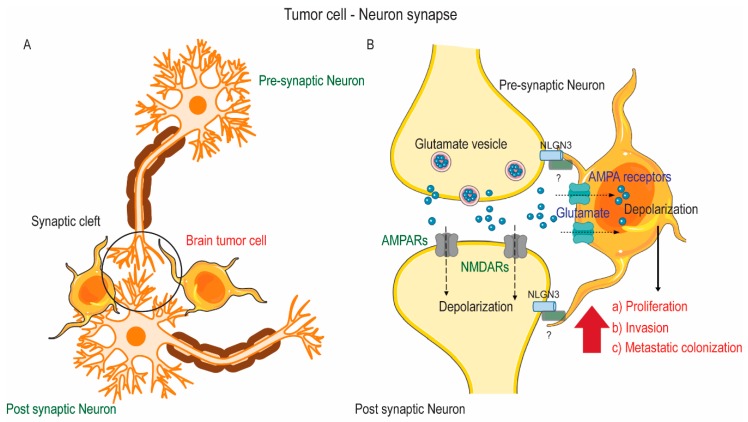
Glioma—Neuron crosstalk. (**A**) Glioma cells form synaptic connections with neurons at glutamatergic synapses. (**B**) Brain tumor cells upregulate glutamate receptor (including AMPA family of receptors) and post-synaptic structural genes (including neuroligin-3) to establish synaptic connections. Electrochemical stimulation of neurons results in release of glutamate in the synaptic cleft, which rapidly depolarizes glioma. This drives an influx of Ca^2+^ ions, which promotes tumor cell proliferation and increases invasiveness and metastatic colonization of glioma cells. Abbreviations: AMPA, α-amino-3-hydroxy-5methyl-4-isoxazole propionic acid; NMDA, N-methyl-D-aspartate; NLGN-3, Neuroligin-3.

Brain tumors act as ‘glutamine traps’ by successfully competing for the glutamine recycled by astrocytes in the brain tumor microenvironment. Tumor cells achieve this by upregulating expression of glutamine and glutamate importers. Several human glioma cell lines are shown to upregulate expression of ASCT2 (SLC1A5), the principal glutamine importer, which drives rapid glutamine uptake in tumor cells [36,37]. Gliomas that are unable to synthesize glutamine de novo can express sodium-coupled amino acid transporters like SNAT3 and SNAT-5 [38,39,40]. These transporters can mediate bi-directional transport of glutamine. Indeed, higher expression of SNAT-3 in some GBMs is associated with increased malignancy [38]. Similarly, brain tumors upregulate expression of NMDA, AMPA, and mGluRs [41,42]. Electrophysiological studies have shown that these tumor cell glutamatergic receptors are functionally similar to those in neurons [43]. Activation of these receptors in glioma cells results in an influx of Ca^2+^ ions, which supports tumor growth and invasion [41]. Interestingly, recent studies report that glioma cells form functional, excitatory synapses with neurons (Figure 2) [44,45,46]. They show that glioma cells, like neurons, express genes encoding glutamate receptors and establish a synaptic network using microtubes. Indeed, stimulation of neurons proximal to these tumor cells produced a depolarizing current in some glioma cells, which increased their proliferation and invasiveness [44,45]. Crucially, this phenomenon is shown to be mediated by a distinct class of AMPA glutamate receptors and pharmacological blockade of these receptors inhibited tumor growth, invasion, and enhanced the susceptibility of gliomas to chemotherapeutic agents [41,42,44]. Rapidly proliferating glioma cells produce high levels of reactive oxygen species (ROS), which leads to increased oxidative stress. As a defense mechanism, brain tumor cells can increase GSH synthesis by upregulating SXC activity [47,48]. Glutathione prevents ROS-mediated cell death and therefore promotes tumor cell survival [49]. Importantly, high levels of GSH also correlate with treatment resistance in gliomas and other cancers [50,51]. Thus, brain tumor cells can hijack glutamine and glutamate transporters to meet their increased metabolic needs to sustain proliferation.

#### Transcriptional Control of Glutamine Metabolism

Several tumor-driving oncogenes, signaling pathways, and tumor suppressors have been implicated to transcriptionally regulate glutamine metabolism in cancer. c-MYC is one such principal regulator. It binds to the promoters of high-affinity glutamine importers, such as ASCT2 (SLC1A5) and SN2 (SLC38A5), resulting in increased expression and a subsequent increase in glutamine uptake [52]. Additionally, c-MYC is also shown to promote transcription of glutaminase by repressing mir23a and mir23b in prostate cancer cells [53]. While in melanomas, it is shown to enhance glutamine-derived nucleotide biosynthesis by upregulating expression of rate-limiting enzymes in deoxyribonucleotide (dNTP) metabolism [54]. Mutations in the PTEN gene results in activation of the phosphatidyl inositol kinase/protein kinase B (PI3K)/Akt/mTOR) pathway and is universally associated with poor prognosis in glioma patients [55,56]. PTEN is shown to regulate glutamine metabolism by modulating expression of glutaminase (GLS) in a PI3K/Akt pathway-dependent manner [57]. Interestingly, the PI3K/Akt axis is also shown to drive production of glutathione (GSH) by promoting stability of nuclear factor-like 2 (NRF2, a key regulator of redox homeostasis ) in breast cancers [58,59]. The mTORC1 complex, downstream of PI3K/Akt pathway, drives expression of glutamate dehydrogenase (GDH) by transcriptionally repressing sirtuin-4 (SIRT-4) [60]. Notably, tumor suppressors such as p53 and the retinoblastoma (Rb) protein are shown to promote glutaminolysis by driving expression of glutaminase–2 (GLS2) and ASCT2, respectively [61,62]. Under nutrient-limiting conditions, p53 is shown to upregulate expression of the glutamate/aspartate transporter (SLC1A3) in colorectal cancer cells, which supports utilization of aspartate in the absence of glutamine [63]. Interestingly, restoring p53 function in pancreatic cancer cells resulted in accumulation of α-ketoglutarate (α-KG), which further lead to activation of tumor cell differentiation programs [64]. Thus, these studies highlight key mechanisms underlying the transcriptional control of glutamine metabolism.

## 3. Functional Roles of Glutamine

### 3.1. Glutamine-Derived α-Ketoglutarate can Function as a TCA Cycle Anaplerotic Substrate and Regulate the Epigenome

Glutamine-derived α-ketoglutarate can serve as a TCA cycle anaplerotic substrate in many cancer cells through oxidative carboxylation. Glutamine oxidation can result in production of three NADH molecules and one FADH_2_ (flavin adenine dinucleotide) molecule which creates the electrochemical gradient needed for ATP production. Cancer cells predominantly utilize glucose as the principal source of carbon for lipid and fatty acid synthesis, and its disruption is shown to hinder tumor formation [65,66]. Glucose-derived carbons can be directed to synthesize citrate, which is then metabolized by ACLY into the acetyl-coA pool needed for lipid biosynthesis. However, cells under hypoxia or cells with mitochondrial defects begin to utilize glutamine as the source of carbon for making acetyl-coA [67,68]. However, it is noteworthy that some GBMs with a mesenchymal phenotype (negative for CD133) exhibit higher glutamine utilization, and inhibiting glutamine metabolism delayed tumor growth in vivo [69]. Glutamine-derived α-ketoglutarate is converted to citrate by isocitrate dehydrogenase (IDH) coupled with NADPH consumption in a reverse process called reductive carboxylation [70]. A shift in the ratio of intracellular α-ketoglutarate to citrate levels can trigger reductive carboxylation [71,72].

Metabolic pathways can directly regulate the epigenetic state of the cell. For example, glucose derived acetyl-CoA serves as an acetyl donor for histone acetyl transferases to acetylate histones. Similarly, glutamine-derived α-ketoglutarate can also regulate the epigenome, as evidenced in IDH1/2 mutant gliomas, as it serves as a co-substrate for a class of dioxygenase enzymes. This includes Jumonji C domain-containing histone demethylases and TET family DNA demethylases, all of which are crucial for regulating gene expression. Indeed, the ratio of α-KG and succinate, a by-product of α-KG-mediated demethylation, dictates pluripotency in embryonic stem cells [73]. IDH1/2 mutations are observed in >70% of grade II and grade III gliomas and more than 90% of secondary glioblastomas [74,75]. Mutant IDH1/2 catalyze the generation of the oncometabolite D-2-hydroxyglutarate (D-2HG) from α-ketoglutarate [68,76]. Notably, in vivo tracing studies using hyperpolarized glutamine have shown that glutamine carbons gives rise to D-2HG in IDH1/2 tumor cells [77]. D-2HG is structurally similar to α-ketoglutarate and inhibits α-KG-dependent enzymes, including histone and DNA demethylases [78,79]. Inhibition of these enzymes by D-2HG results in genome-wide epigenetic alterations [68,76], including high H3K9 trimethylation and CpG island methylation [80,81,82].

### 3.2. Glutamine can Serve as a Nitrogen Donor for Nucleotide Synthesis

In addition to serving as a major carbon source for driving TCA cycle anaplerosis and macromolecule synthesis, glutamine is a critical nitrogen donor for de novo nucleotide biosynthesis. The amide group (containing γ-nitrogen), resulting from the deamination of glutamine to glutamate, enables production of nucleotides, amino sugars, and NAD+ co-factors. Cancer cells utilize the activity of different enzymes to mediate nucleotide synthesis from glutamine. Purine synthesis involves utilization of two glutamine nitrogens for production of inosine monophosphate (IMP), which further gives rise to adenosine and guanosine monophosphate (AMP and GMP) [83,84]. 5-phoshoribosyl α-pyrophosphate, a product of the pentose phosphate pathway, is converted into phosphoribosyl-β-amine with addition of amide group from glutamine by phosphoribosyl pyrophosphate amidotransferase (PPAT). This serves as an important intermediate in purine synthesis [85]. Likewise, initiation of pyrimidine synthesis involves the condensation of glutamine-derived nitrogen with bicarbonate and ATP [83,84]. Carbamoyl phosphate synthetase II (CPSII) drives this rate limiting step to generate carbamoyl phosphate. Finally, cytidine triphosphate synthetase (CTPS) utilizes another glutamine-derived amide group for conversion of uridine triphosphate (UTP) into cytidine triphosphate (CTP). It is noteworthy that expression of enzymes that mediate nucleotide synthesis from glutamine, such as CPSII, PPAT etc., are facilitated by oncogenic drivers like c-Myc and mutant p53 [86,87]. Indeed, a higher expression of these enzymes positively correlates with increased proliferation in tumors [53,88]. The importance of glutamine-derived nucleobases is underscored by the ability of exogenous nucleotides to rescue glutamine-deprived cancer cells from undergoing cell cycle arrest [89,90,91]. More importantly, primary lung tumors are shown to utilize glutamine for nucleotide synthesis when cultured ex vivo [92].

### 3.3. Glutamine as a Source of Non-Essential Amino Acids

Cancer cells have greater energy needs to sustain continuous proliferation. Their high metabolic demands make them dependent on glutamine as a source of non-essential amino acids (NEAAs) [93]. They rely on the activity of GLS to maintain a high ratio of glutamate to α-ketoglutarate, which is essential for production of NEAAs. Interestingly, proliferating cells are shown to be metabolically distinct when compared to quiescent cells in their capacity to catabolize glutamate for NEAA synthesis [94,95]. To this end, the nitrogen at the α-position on the glutamate carbon chain is transferred to different α-ketoacids by aminotransferase enzymes. Aspartate, alanine, ornithine, and phosphoserine function as nitrogen acceptors. Importantly, the inherent inefficiency of the aspartate transporter necessitates the production of a majority of intracellular aspartate from glutaminolysis [96,97,98]. Indeed, it has been shown that cytosolic aspartate is a key determinant of cancer cell survival under glutamine-deprived conditions [99]. Moreover, expression of SLC1A3 (glutamate-aspartate transporter) induced by p53 and YAP/TAZ is also shown to promote tumor growth under glutamine-limiting conditions [63,100,101]. Collectively, these studies highlight the role of glutamine-derived aspartate in tumor growth and survival.

Additionally, alanine and phosphoserine can also be synthesized from glutamine-derived nitrogens by the enzymes alanine aminotransferase (GPT) and Phosphoserine aminotransferase (PSAT). Intracellular alanine concentration measured from prostate cancer biopsies showed a positive correlation with patient survival [102]. Notably, these glutamine-utilizing transaminases serve as metabolic vulnerabilities in YAP/TAZ activated breast cancers, as they utilize PSAT1 and glutamic-oxaloacetic transaminase (GOT1) to promote glutamine dependence and drive tumor growth [103]. Repression of PSAT1 decouples glutamine-driven anaplerosis from NEAA synthesis, resulting in growth arrest in vitro and in vivo. Similar to alanine and phosphoserine, asparagine is important for tumor cell survival [95,104]. Asparagine synthetase (ASNS) catalyzes synthesis of asparagine from glutamine and aspartate. Glutamine deprivation leads to an accumulation of uncharged t-RNAs, which induces endoplasmic reticulum (ER) stress. Asparagine is shown to have a protective effect on glioblastoma (GBM) tumor cells under such glutamine-limiting conditions by mediating ATF (AMP-dependent transcription factor)-dependent stress responses, which prevents cell death [105]. Further, glutamine is an important source for synthesis of polyamines and glycopolymers, and can also serve as a precursor for NAD synthesis [106,107].

### 3.4. Role of Glutamine in Maintaining Redox Homeostasis

Oxidation of glutamine in the TCA cycle results in production of NADH and FADH_2_ molecules, which generate an electrochemical gradient to produce ATP. Transfer of electrons through the mitochondrial electron transport chain (ETC) by NADH produces reactive oxygen species (ROS). ROS comprises of superoxide (O_2_^−^) anions, hydrogen peroxide (H_2_O_2_), and hydroxyl free radicals. While physiological levels of ROS can be pro-tumorigenic [108], excess ROS results in DNA damage, lipid peroxidation, and protein denaturation [109]. Tumor cells mitigate excess ROS and maintain redox homeostasis principally by glutathione synthesis [110,111]. Glutathione is a tripeptide synthesized from glutamate, cysteine, and glycine that actively scavenges ROS. Glutamine-derived glutamate and cysteine (imported by SXC) is converted into γ-glutamyl cysteine by the enzyme glutamate-cysteine ligase (GCLC) and subsequently into glutathione by glutathione synthetase (GSS). High glutathione levels correlate positively with drug resistance in tumors [112]. Additionally, glutathione serves as a checkpoint for amino acid biosynthesis, and its supplementation can partially overcome effects of amino acid deprivation [113]. Thus, glutamine directly contributes to glutathione biosynthesis by acting as the donor of glutamate, from the GLS reaction, and by enabling uptake of cystine through SXC. Additionally, glutamine produces reducing equivalents by mediating NADPH synthesis through GLUD, oxoglutarate dehydrogenase (OGDH), and malate dehydrogenase (MDH).

### 3.5. Understanding Glutamine Metabolic Heterogeneity in Cancers

Several factors related to heterogeneity in glutamine utilization need to be considered in understanding glutamine metabolism in brain tumors and cancers in general. These include in vitro cell culture conditions, tumor subtype, driver oncogenes, and the tumor microenvironment. One of the major challenges lies in understanding metabolic adaptations when tumor cells are cultured in vitro. For example, there is a shift from oxidative glutamine metabolism to reductive carboxylation when non-small cell lung cancer (NSCLC) cells transition from a monolayer cultures to spheroids [114]. Under monolayer culture, these cells showed abundant glutamine uptake to drive TCA cycle anaplerosis, which is lost under anchorage-independent growth. This shift in glutamine utilization is mediated by cytosolic IDH1 that converts glutamine-derived α-ketoglutarate to citrate along with NADPH in order to mitigate mitochondrial ROS levels. A similar discrepancy in glutamine catabolism was observed when NSCLC cells were transitioned from ex vivo culture to an in vivo environment [92,115]. When cultured in vitro, KRAS-driven NSCLC cells exhibited a strong glutamine dependence for their bioenergetic needs and were susceptible to GLS inhibition. However, this phenotype was lost completely in vivo, as the tumors primarily utilized glucose to drive TCA cycle through upregulation of the enzyme pyruvate carboxylase (PC). Indeed, expression of PC in tumors is a key determinant of glutamine-independent growth [116,117,118]. Similarly, some tumors can utilize glucose to synthesize glutamate de novo, which can then be converted to glutamine by the enzyme glutamine synthetase (GLUL). Such tumors have high GLUL expression and do not depend on exogenous glutamine for their nutrient needs. This is true in some GBM cell lines where it is shown that high GLUL activity drives de novo glutamine synthesis [119,120]. These tumor cells utilize glucose-derived glutamate to synthesize glutamine in a GLS-independent manner, and hence do not respond to glutaminase inhibition. Contrastingly, tumors with low GLUL expression rely exclusively on the exogenous supply of glutamine. They upregulate expression of GLS to effectively drive glutaminolysis [121]. Differences in GLUL expression are also observed in human GBMs where up to 25% patients showed minimal expression (comparable to neurons) and 15% patients showed very high expression (comparable to astrocytes) [120]. Thus, the expression levels of enzymes like GLUL, GLS, and PC can regulate heterogeneity in glutamine metabolism in tumors and could possibly predict response to treatments like glutaminase inhibition.

The metabolic phenotype of the tumors is also influenced by the tissue of origin. This is because different tissues exhibit varying extents of glutamine metabolism in the body, thus determining the levels of exogenous glutamine available for use by tumor cells. For example, kidneys primarily use glutamine to drive anaplerosis and maintain pH balance [122], whereas muscles and adipose tissue largely promote de novo glutamine synthesis [122,123]. Notably, it is also shown that environmental cystine can dictate glutamine dependence and anaplerosis in A549 NSCLC cells [124,125]. Here, cystine in the environment upregulates expression of the xCT, a critical component of SXC, which controls glutamate efflux. The glutamate derived from glutamine is exchanged for cystine through xCT, which decouples glutaminolysis from TCA cycle anaplerosis, resulting in resistance to glutaminase inhibition. Indeed, expression of xCT in human haploid cells favored glucose addiction and antagonized glutamine-driven anaplerosis [126].

An important consideration in understanding glutamine metabolism is in taking into account the tumor microenvironment. Indeed, recent studies have leveraged techniques like mass spectrometry and nuclear magnetic resonance (NMR) to quantitatively measure metabolites in murine pancreatic and lung tumors, and to accurately measure the flux of lactate between tumor cells and the interstitium [127,128]. These techniques can serve as important tools in dissecting the impact of brain tumor microenvironment on glutamine metabolism. In addition to neurons and astrocytes that regulate the synaptic glutamate/glutamine cycle, the brain tumor microenvironment also contains immune cells. Macrophages and microglia constitute the majority of the immune cells in brain tumors, comprising ~30% of the tumor mass [129]. TAMs mediate an immune-suppressive response by secretion of anti-inflammatory cytokines like Interleukins (IL)-4, IL-10, and IL-13 [129]. These secreted cytokines can affect glutamine metabolism, as evidenced in breast cancer cells, where IL-4 secreted from immune cells increased expression of the glutamine transporter, SLC1A5 [130]. Macrophages themselves rely on glutamine to produce cytokines like Tumor Necrosis Factor (TNF), IL-1, and IL-6, and for N-glycosylation of cell surface receptors [131]. Interestingly, TAMs are shown to upregulate enzymes like transaminase (GPT) and GLUL, suggesting a possible tumor promoting role by driving glutamine metabolism [132]. It is important to note that tumor-infiltrating immune cells and proliferating cancer cells can share similar energy demands. For example, T-cells upregulate glycolysis and glutaminolysis to support their energy needs upon immune activation. CD8^+^ T-cells also actively use glutamine to mediate an anti-tumor, cytotoxic response [133,134]. Overall, glutamine metabolism in tumors is regulated by multiple factors that need to be considered before therapeutic targeting of glutamine metabolism including in vitro experimental conditions, driver oncogenes, cell-of-origin, and the tumor microenvironment.

## 4. Metabolic Imaging and Therapeutic Targeting of Glutamine Metabolism

### 4.1. Imaging Glutamine Uptake and Metabolism

Positron emission tomography (PET) imaging is a highly sensitive clinical tool used to assess tumor metabolism in vivo. Labelled radionuclides such as ^11^C or ^18^F undergo a process known as annihilation, resulting in the emission of positrons which can then be detected using a PET scanner. A radiolabeled analog of glucose, 2-[^18^F] fluoro-2-deoxy-D-glucose (FDG) is used extensively in clinical settings, as a surrogate for the Warburg effect, to evaluate tumor metabolism in vivo. FDG import into cancer cells is facilitated by glucose transporters, like GLUT1, where it undergoes phosphorylation by hexokinase to form FDG-6-phosphate (FDG-6-P). However, FDG-6-P can neither be metabolized further nor diffuse out of the cell, resulting in its accumulation inside cancer cells [135]. Thus, FDG-PET is an important tool used in tumor detection and monitoring. FDG-PET can also serve as a clinical readout for treatment response. However, despite its widespread clinical use, FDG-PET has limitations in evaluating brain tumors, largely because of high background from the normal glucose uptake in the brain resulting in a low tumor-to-background ratio [136,137].

Use of radiolabeled glutamine circumvents this problem, as brain tumors show preferential uptake of glutamine compared to surrounding brain tissue. This can be achieved by using radionuclides like ^18^F or ^11^C [138,139,140]. ^11^C-glutamine is actively transported and incorporated into cellular protein after incubation for 30 min by 9L and SF-188 glioma cells [141]. However, it has a very short half-life of ~20 min [141]. ^18^F-labeled glutamine has a much longer half-life of 110 min, making it an ideal PET agent. Tumor cells that overexpressed SLC1A5 displayed higher uptake and retention of the ^18^F-labeled [2S,4R] stereoisomer of glutamine [45,46]. Animal glioma models and human glioma patients show high uptake of ^18^F-FGln compared to normal brain tissue in vivo, resulting in distinct tumor delineation [142,143]. ^18^F-FGln has a high tumor-to-background signal ratio ranging from 4:1 to 6:1 compared to ^18^F-FDG (~1:1). Furthermore, disruption in the blood brain barrier (BBB) following neuro-inflammation did not increase ^18^F-FGln uptake in the brain, underscoring its specificity to tumors. Interestingly, ^18^F-FGln uptake is reduced in gliomas treated with chemotherapy or radiation, highlighting the potential of ^18^F-FGln-based PET imaging as a tool to monitor treatment response in patients. The recently completed clinical trial (NCT01697930) measuring tumor glutamine uptake using ^18^F-FGln demonstrates its feasibility as a radiological probe with no adverse effects in patients [144]. ^18^F-FGln is able to successfully image several cancers with glutamine hypermetabolism, thus validating its use as a potential tool for targeted radiotracer imaging [145,146,147]. (S)-4-(3-[^18^F]Fluoropropyl)-L-glutamic acid (^18^F-FSPG) is a glutamate analogue that can be used as a radiopharmaceutical to measure the activity of the glutamate/cystine antiporter, xCT [145,146]. ^18^F-FSPG is specifically transported by xCT and provides excellent tumor visualization with high contrast (tumor/brain signal ratio: 32.7) in glioblastoma (GS9L) animal models [145]. More importantly, a completed clinical trial (NCT01186601) for evaluation of ^18^F-FSPG-based PET imaging in brain tumor patients successfully validated the use of the tracer as an imaging tool [145].

Magnetic resonance spectroscopy (MRS) is a modality that measures metabolite concentrations in vivo. MRS utilizes specific radio-frequency signals emitted from the nuclear spins of ^1^H, ^31^P, and ^13^C to detect cellular metabolites in tumors and the microenvironment [148]. ^1^H-MRS imaging is clinically used to detect various metabolic spectra within human tumors with high levels of sensitivity [149]. Both glutamine and glutamate have similar spectra and are evaluated collectively as Glx. MRS is an important tool utilized for assessing Glx levels in both animal models and in patients with brain tumors [150]. It has been successfully used in gliomas, meningiomas, and medulloblastomas, and can inform tumor subtype, grade, and invasion [151,152,153].

Glutamine-based PET imaging and ^1^H-MRS imaging are both excellent tools to evaluate glutamine hypermetabolism and estimate global levels of metabolites in tumors. However, these tools are limited by their inability to delineate the metabolic fate of glutamine in tumor cells. This limitation can be overcome by isotope labeling of glutamine, which enables detection of the tracer in tumor tissues along specific metabolic pathways [154]. ^13^C labelling aids in tracing the fate of glutamine-derived carbons, and ^15^N labeling helps follow glutamine nitrogens along metabolic pathways. Tracing the fate of glutamine in vivo involves exogenously injecting labeled glutamine using isotopic labeling followed by harvesting tumor tissues to assess isotope incorporation in various metabolites. The sensitivity of ^13^C/^15^N labeling in vivo can be enhanced 10,000-fold through hyperpolarization [155]. In this technique, the tracer is exposed to microwaves at low temperatures prior to administration and scanning. This enables real-time detection of metabolic fluxes with enhanced sensitivity [156]. Hyperpolarized ^13^C-labeling of glutamine has shown great promise in certain liver cancer models [157,158,159]. This technique has proven to be particularly useful in mutant IDH1-driven tumors, where it issued to quantify production of the oncometabolite D2-HG in vivo [77]. Furthermore, isotope labeling can be used to demonstrate the heterogeneity in glutamine metabolism under different conditions. However, it is noteworthy that hyperpolarization is limited by the shorter half-life of the imaging agent, thereby drastically reducing the time window for imaging. Hyperpolarized ^13^C spectra has a lower gyrometric ratio than a proton, which results in lesser artifacts, but it requires higher imaging gradient amplitudes to achieve similar spatial resolution [160].

Currently, magnetic resonance imaging (MRI) and ^18^F-FDG-based PET and computed tomography (CT) are routine imaging modalities for the clinical management of brain tumors. However, as highlighted earlier, these techniques show limited sensitivity and specificity to tumor tissue. Thus, glutamine metabolism-based modalities can potentially define a new paradigm of brain-tumor imaging.

### 4.2. Therapeutic Targeting of Glutamine Metabolism in Cancer

The pleiotropic role of glutamine in regulating critical cellular functions such as energy production, macromolecular biosynthesis, cell signaling, and redox homeostasis has made it an attractive and druggable therapeutic target (Table 1). Tumor cells achieve high intracellular concentrations of glutamine primarily through upregulation of glutamine transporters including SLC1A5 and SLC7A5 [161]. Known inhibitors of SLC1A5 such as 6-diazo-5-oxo-l-norleucine (DON), Benzylserine, and L-γ-glutamyl-p-nitroanilide (GPNA) have been shown to effectively suppress tumor growth in vitro and in vivo [161,162]. Pharmacological blockade of SLC1A5 and SLC7A5 using a newly designed small molecule antagonist, V-9302, elicited a marked anti-tumor response in pre-clinical tumor models [163]. An analog of DON with masked carboxylate and amine functionalities, JHU-083, was recently shown to have increased bioavailability, resulting in higher cell kill and decreased growth of Myc-driven medulloblastomas in vivo [164]. Further, inhibitors such as bis-2-(5-phenylacetamido-1,2,4-thiadiazol-2-yl)ethyl sulfide (BPTES), CB-839, and compound 968 represent a unique class of drugs developed to target tumor-specific isoforms of glutaminase (GLS) [165,166,167]. Compound 968 specifically inhibits GAC, a shorter kidney-type isoform of GLS, by repressing Rho GTPases [166]. In contrast, BPTES and CB-839 both allosterically inhibit tetramerization of GLS [165,167,168], and have shown great promise as targeted therapies for glutamine-addicted tumors [165,169]. CB-839, an improved derivative of BPTES with an IC_50_ < 50 nM against human GLS, is currently in Phase I clinical trials. The initial results of these trials for hematological malignancies (NCT02071927 and NCT02071888) report a high tolerance for CB-839 in patients, along with robust inhibition of GLS and subsequent reduction in blast counts [170]. However, it is important to note that inhibition of glutaminase in tumors may result in development of compensatory metabolic networks [171]. This may necessitate the design of rational combinatorial strategies that can target multiple metabolic nodes. Similarly, inhibitors of glutamate dehydrogenase (GLUD) such as Epigallocatechin gallate (EGCG) and R162 are shown to be effective in hindering tumor proliferation by disrupting the anaplerotic use of glutamine in the TCA cycle [172,173]. It is noteworthy that glutamine metabolism is essential for endothelial cell function [174] in tumors and glutamine deprivation or disruption of glutaminase impairs angiogenesis in vivo [175]. This suggests that inhibition of glutamine metabolism in vivo can disrupt blood supply to tumors and potentially augment treatment response. These promising findings strongly emphasize that targeting glutamine metabolism can be an attractive therapeutic strategy.

## 5. Conclusions

**Figure 3:**
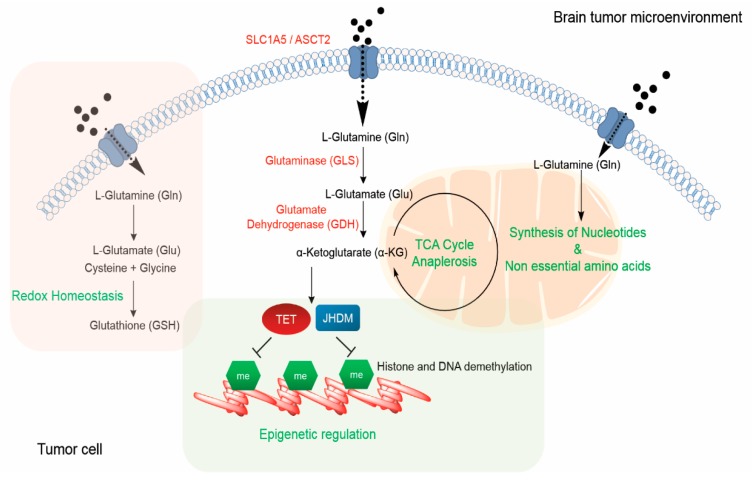
Functional roles of glutamine in regulating tumor progression. Glutamine from the brain tumor microenvironment is taken up by tumor cells via ASCT2/SLC1A5 and converted into glutamate by the enzyme, glutaminase (GLS). Glutamate, along with cysteine and glycine, can be utilized by the tumor cell to synthesize glutathione (GSH), which is pivotal for maintaining redox homeostasis. Glutamate dehydrogenase (GDH) converts glutamate to α-ketoglutarate (α-KG), which drives TCA cycle anaplerosis. Glutamine metabolism can influence the tumor cell epigenome and methylome by modulating the levels of α-KG, which is an important co-factor for DNA (TET) and histone demethylases (JHDM). Additionally, glutamine plays a key role in mediating synthesis of nucleotides and other non-essential amino acids. Abbreviations: TCA, tricarboxylic acid; TET, Tet methyl cytosine dioxygenase; JHDM, Jumonji domain-containing histone demethylase.

Glutamine metabolism can regulate multiple pathways including macromolecule synthesis, energy production, epigenetic regulation, and redox homeostasis in brain cancer cells (Figure 3), making it an attractive therapeutic target. However, it is important to note that glutamine metabolism depends on a multitude of factors that can give rise to heterogeneity including cells-of-origin, tumor-intrinsic genetic aberrations, and the tumor microenvironment, all of which can influence glutamine requirements of brain tumor cells in vivo. Variation in the tumor metabolic state, influenced by experimental systems, constitutes an additional layer of complexity. Further research to improve cell culture systems that can better reflect the in vivo state would be critical to better understand glutamine metabolism. Nevertheless, with the advancing technologies, including understanding the tumor microenvironment and newer tools to assess glutamine metabolism in vivo, bear promise to inform brain tumor monitoring and treatment.

## Figures and Tables

**Table 1:** Therapeutic strategies targeting glutamine metabolism in tumors.

Class	Drug	Status
Glutamine mimics	DON JHU–083 [164] Azaserine Acivicin	Limited by off target toxicity
Glutaminase (GLS) inhibitors	Compound 968 [166] CB-839 [167] BPTES [165,168]	Preclinical tool Successful in Phase I clinical trials [NCT02071927, NCT02071888] Preclinical tool
SLC1A5 inhibition	V-9302 [163] Benzylserine GPNA γ-FBP	Effective, preclinical tools
Glutamine depletion	L–Asparginase	Clinically used for hematological malignancies Limited by toxicity
Glutamate dehydrogenase (GLUD) inhibitors	EGCG R162	Preclinical tool compounds
Aminotransferase inhibitors	AOA	Used for tinnitus treatment Limited by toxicity
SLC7A11 or xCT inhibitors	Sulfasalazine Erastin	Pre-clinical tools Potent inducers of ferroptosis
